# *Nigella sativa* powder for helicobacter pylori infected patients: a randomized, double-blinded, placebo-controlled clinical trial

**DOI:** 10.1186/s12906-023-03955-4

**Published:** 2023-04-17

**Authors:** Hedieh Yousefnejad, Farzaneh Mohammadi, Mahvash Alizadeh-naini, Najmeh Hejazi

**Affiliations:** 1 Department of Clinical Nutrition, School of Nutrition and Food Sciences, Shiraz University of Medical Sciences, Shiraz, Iran; 2 Student Research Committee, Shiraz University of Medical Sciences, Shiraz, Iran; 3 Department of Internal Medicine, School of Medicine, Shiraz University of Medical Sciences, Shiraz, Iran; 4 Nutrition Research Center, Department of Clinical Nutrition, School of Nutrition and Food Sciences, Shiraz University of Medical Sciences, Shiraz, Iran

**Keywords:** *Nigella sativa*, *Helicobacter pylori*, Appetite, Ghrelin, Integrative medicine, Herbal medicine

## Abstract

**Objective:**

This double-blind, placebo-controlled, clinical trial was conducted to define the effects of Nigella sativa (*N. Sativa*) powder plus conventional medical treatment of *Helicobacter pylori* (*H. pylori*) on serum ghrelin level and appetite in *H. pylori*-infected patients.

**Methods:**

In the present study, 51 *H. pylori*-positive patients were randomly allocated to treatment (n = 26) or placebo (n = 25) groups. They received 2 g/day *N. Sativa* with quadruple therapy or 2 g/day placebo plus quadruple therapy for 8 weeks. The serum level of ghrelin was assessed before and after the intervention. Appetite was evaluated at the onset and at the end of the intervention.

**Results:**

At the end of the study, the appetite of the treatment group improved significantly compared with the placebo group (P = 0.02). Statistically, the difference in serum ghrelin levels between the study’s groups was insignificant (P > 0.05).

**Conclusion:**

Supplementation with *N. Sativa* powder may be a beneficial adjunctive therapy in *H. pylori*-infected patients.

**Trial registration:**

This study was registered in the Iranian Registry of Clinical Trials (IRCT20170916036204N7) on 08/08/2018.

## Introduction

Each microbe needs to be able to best exploit the host environment and protect itself from deleterious factors. This is especially the case for a gram-negative microaerophilic bacterium that is named *Helicobacter pylori* (*H. pylori)* [1]. *H. pylori* penetrates the gastric mucus layer and secretes some virulence factors such as lipopolysaccharides (LPS), cytotoxin-associated gene A (cagA), and vacuolating cytotoxin A (vacA) into the host cells’ cytoplasm and eventually leads to inflammation and harm to the gastric epithelial cells [2]. *H. pylori* infection is implicated in gastritis, dyspepsia, peptic ulcer, and gastric cancer [1]. Worldwide, 50.8% of the people in developing countries and 34.7% of those in developed countries are infected with this infection [3].

Ghrelin is an appetite-stimulating peptide with 28 amino acids which is produced in the gastric oxyntic gland. This neuroendocrine hormone has an essential part in the regulation of energy homeostasis, fat storage, and increase of appetite [4]. Some previous studies have reported the relationship between *H. pylori* infection and eradication and the level of plasma ghrelin. In some of these studies, plasma ghrelin levels increased or decreased after the *H. pylori* infection is eradicated while in some others no relationship was observed. On the other hand, this relationship has remained controversial [5, 6].

Herbal medicine has been effective and safe in the treatment of many gastrointestinal disorders (GI), including ulcerative colitis, functional dyspepsia (FD), diarrhea, stomachache, etc. [7–9].

*Nigella sativa* (*N. Sativa*) as a member of the *Ranunculaceae* family is mostly cultivated in the Middle Eastern Mediterranean region, Syria, Turkey, Saudi Arabia, India, and southern Europe [10]. It is one of the most beneficial medicinal herbs in traditional medicine and has been widely investigated in recent years due to its effects in the treatment of many diseases and symptoms such as gastrointestinal disorders, jaundice, anorexia, diarrhea, liver disease, fever, dizziness, inflammation, diabetes and overall for promotion of human health [11, 12]. The seeds of *N. Sativa*, generally recognized as black cumin or black seed, are the source of biologically active components such as thymol, dithymoquinone, thymoquinone, nigellicine, nigellidine, etc. which are synergistically responsible for beneficial health effects [13, 14]. Anti-bacterial properties of *N. Sativa* and its bioactive ingredients have been examined in several studies. Some clinical trials demonstrated that ingestion of *N. Sativa* seed powder or oil could eradicate *H. pylori* infection and improve symptoms of dyspepsia in the infected patients [10, 15, 16]. Also, in-vitro studies revealed that *N. Sativa* extract could inhibit the growth of 100% of all the *H. pylori* strains in 60 min [17] and its essential oil; also, thymoquinone and hydrothymoquinone have shown strong anti-bactericidal properties against some Gram-positive and Gram-negative bacteria [18–21].

Based on our knowledge, this clinical trial is the first study to examine the effects of adding *N. Sativa* powder to a quadruple therapy on serum ghrelin levels and appetite in Helicobacter pylori-positive patients.

## Materials and methods

### Study design

The present randomized, double-blinded, placebo-controlled clinical trial was executed according to the declaration of Helsinki and good clinical practice guidelines. The ethics committee of Shiraz University of Medical Sciences, Shiraz, Iran permitted the protocol (IR.SUMS.REC.1396.98). This study was also registered in the Iranian Registry of Clinical Trials (IRCT20170916036204N7) and was conducted between October 2017 and September 2018. Patients who were referred to the gastroenterology clinic of Motahari polyclinic, Shiraz University of Medical Sciences, Shiraz, Iran, and met the inclusion criteria were comprised in the present study.

### Participants

**Fig. 1:**
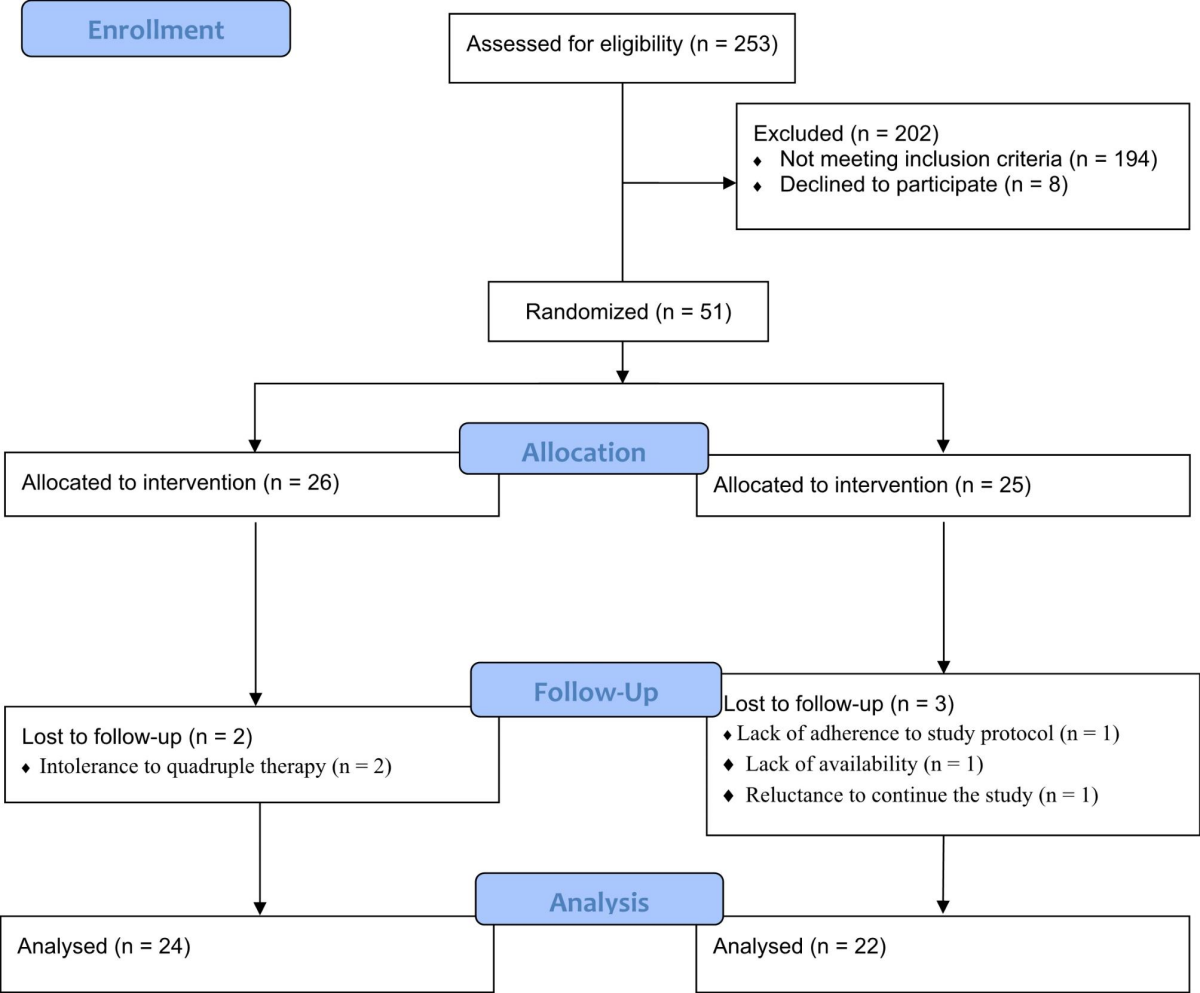
Consolidated standards of reporting trials (CONSORT) flow diagram

After screening 253 patients, 51 eligible patients (16 males and 35 females) aged 18–65 years old with a positive test of *H. pylori* by UBT (Urea Breath Test), stool antigen test, or endoscopy biopsy who were willing to participate in the trial were enrolled in the study (CONSORT diagram, Fig. 1). The exclusion criteria of the study included those with (1) having chronic and inflammatory diseases such as liver disease, inflammatory bowel disease, kidney disease, diabetes mellitus, lung disease, systemic inflammation, HIV, cancer, etc.; (2) having a history of gastric surgery or gastric cancer; (3) suffering intense gastritis and active GI bleeding; (4) taking bismuth or any antibiotics during the previous 6 weeks; (5) taking all types of medication for *H. pylori* before the study; (6) drinking alcohol or using narcotic; (7) eating a specific diet such as vegetarian diet or taking medicinal herbs or being on weight reduction diets; (8) body mass index (BMI) more than 30 kg/m^2^, and (9) being pregnant and lactating. The aim of the study was explained to all the subjects and informed written consent was obtained.

### Sample size

The sample size was determined according to variations in the serum ghrelin level based on the outcomes of a previous study [4]. Regarding a mean difference of 10.63, σ = 10.23, and R = 2, and a probability of 80% with the predetermined level of α = 0.05, a sample size of 11 participants per group was calculated. According to the probable attrition in the sample, the final sample size was considered 30 patients in each group.

### Randomization

The participants were equally and randomly allocated to the intervention or control groups by block randomization technique via random allocation software [22]. Allocation concealment was done by an outsider person. He prepared 2 blocks of random blocks, then randomly identified the sequences and groups them in a closed envelope, named A and B. After the individuals entered the study, the sealed envelopes containing the assigned group of participants opened for each participant based on the sequence determined by the person outside the study.

### Interventions

The patients were assigned to receive 2 gr N.sativa powder (2 capsules of 500 mg twice a day, 30 min after the meal) or 2 gr colored pharmaceutical starch powder as the placebo (2 capsules of 500 mg twice a day, 30 min after the meal) in the intervention and control groups, respectively for 8 weeks. The dose of *N.sativa* powder was determined based on a previous study [23]. Simultaneously with this intervention, all the patients underwent quadruple therapy for 2 weeks. This therapy included 40 mg of omeprazole 2 times a day, 240 mg of bismuth subcitrate 2 times a day, 500 mg of metronidazole 2 times a day, and 1000 mg of amoxicillin 2 times a day.

### Preparation of placebo and ***N. Sativa*** capsules

For the preparation of the placebo, the pharmaceutical starch powder was bought by the Faculty of Pharmacy (Shiraz, Iran) and dyed with a dark brown allowed edible dye. After microbial evaluation of four samples of *N. Sativa* seed (in nutrition broth media) in the laboratory of the school of nutrition and food sciences, Shiraz, Iran, a suitable sample with the minimum microbial growth was carefully chosen for the preparation of supplements. Then, it was purchased for the required amount from an authentic local market and ground in the school of pharmacy (Shiraz, Iran). Encapsulation of the powders in the 500-milligram soft gel capsules provided by Arian Salamat Company (Tehran, Iran) was done in the school of pharmacy. All capsules were stored in a dry and dark place at room temperature (below 25 °C) before the beginning of the study.

### Blinding

Because of the completely similar color, smell, and size of the *N. Sativa* powder and placebo capsules, the participants, researcher, and gastroenterology clinic staff were blind to group allocation.

### Questionnaires

At the baseline of the study, a demographic information questionnaire including age, sex, educational level, physical activity, weight, and BMI was completed for all the participants. BMI was calculated by dividing the weight (kg) by height^2^ (m). In the beginning and at the end of the intervention, the appetite questionnaire CNAQ (the Council on Nutrition Appetite Questionnaire) including 7 questions was accomplished for each patient. Each question had a score of 1 to 5. (1 for the most negative rate and 5 for the most positive rate) [24].

### Blood sampling and biochemical measurement

At the beginning and the end of the intervention, after 10–12 h of fasting, a 7-milliliter venous blood sample was collected from each individual and centrifuged (2000 g for 10 min) to isolate the serum. The samples were kept at -80° C until biochemical analysis.

In the present study, the primary outcome was the determination of variations in the serum ghrelin concentration from the baseline to the end of the intervention. Serum ghrelin concentration was measured by ELISA kits (Crystal day, China, cat. No: E3091Hu) before and after the intervention.

### Statistical analysis

Data were analyzed by statistical package for the social sciences (SPSS) software version 22 (SPSS, Inc., Chicago, USA). The normality of the data distribution was evaluated by the Shapiro-Wilk test. Comparing the categorical variables between the groups performed by the Chi-square test. Within-group and between-group comparisons were done by the Wilcoxon and the Mann-Whitney u-tests, respectively. The descriptive variables were reported by means ± SD or median (IQR) for quantitative data and number (percentage) for qualitative ones. To determine the reliability of the Persian format of CNAQ, the internal consistency of the questions was tested by Cronbach’s Alpha. Test-retest was used to determine the repeatability of the questions (by using Pearson’s correlation analysis). The content validity of the questions was evaluated by 15 nutritionists and the content validity ratio (CVR) and content validity index (CVI) of each question were measured. A P-value < 0.05 was considered statistically significant.

## Results

### Participants and baseline characteristics of participants

A total of 51 eligible participants were recruited for the present study from October 2017 to September 2018. Finally, 46 subjects (*N. Sativa* group = 24, placebo group = 22) accomplished the clinical trial (Fig. 1). Two patients in the *N. Sativa* group and 3 in the placebo group were excluded from the trial due to their intolerance to quadruple therapy, reluctance to continue the study, lack of adherence to the study protocol, and lack of availability. The patients who ended the study consumed at least 95% of the capsules.

Baseline demographic and anthropometric characteristics of the patients are presented in Table 1, showing that there was no significant difference between the study groups (P > 0.05).

**Table 1:** Baseline demographic and anthropometric characteristics of the participants in the *N. Sativa* and Placebo groups

variables	N. Sativa group (n = 26) number	Placebo group (n = 25) number	P-Value
Age (year)^c^	40.61 ± 10.65	40.12 ± 10.77	0.87^a^
Sex N (%)			0.36^b^
female	16 (61.5%)	19 (76%)	
male	10 (38.5%)	6 (24%)	
Menopausal status N (%)			0.09
premenopausal	8 (30.8%)	15 (60%)	
postmenopausal	8 (30.8%)	4 (16%)	
Physical activity N (%)			0.33
daily	3 (11.5%)	3 (12%)	
weekly	10 (38.5%)	4 (16%)	
monthly	1 (3.8%)	1 (4%)	
sometimes	12 (46.2%)	17 (68%)	
Weight (kg)^c^	71.06 ± 12.20	70.89 ± 11.17	0.96^a^
BMI (kg/m^2^)^c^	24.65 (6.39)	26.05 (6.59)	0.34^a^

^a^ Between-group comparison (Mann-Whitney u-test)

^b^ Between-group comparison (Chi-square test)

^c^ These variables are reported as mean ± SD or Median (IQR)

### Serum level of ghrelin

The comparison of the serum level of ghrelin between the study groups before and after the intervention is presented in Table 2. As shown in this Table, the within-group and between-group changes in ghrelin level were not statistically significant (P > 0.05).

**Table 2:** The comparison of the serum level of ghrelin between the two groups before and after the intervention

Variables	*N.sativa* group (n = 24)	Placebo group (n = 22)	P-value^b^
Ghrelin (pg/ml)			
Before	388.20 (2569.13) ^*^	415.75 (620.63)	0.86
After	435.90 (2904.55)	397.55 (670.43)	0.61
Mean change	24.85 (207.83)	2.90 (199.75)	0.42
P-value^a^	0.21	0.96	

Note: Distribution of the variable was not normal

* Median (IQR)

^a^ Within-group comparison (Wilcoxon test)

^b^ Between-group comparison (Mann-Whitney u-test)

### Appetite

Table 3 shows the comparison of the patients’ appetite at the beginning and at the end of the study in the *N. Sativa* and placebo groups. According to this table, appetite improved significantly in the intervention group compared to the placebo at the end of the study (P = 0.02). Also, the within-group assessment indicated a significant improvement in appetite in the intervention group (P = 0.01), but no significant changes were observed in the control group (P > 0.05) during the study (Table 3).

**Table 3:** The comparison of the patients’ appetite between the two groups at the baseline and end of the intervention

Variables	*N.sativa* group (n = 24)	Placebo group (n = 22)	P-value^b^
Appetite			
Before	26.0 (3.75)	26.0 (5.0)	0.61
After	27.0 (3.0)	25.5 (5.0)	0.03
Mean change	2.5 (4.0)	0.0 (4.0)	0.02
P-value^a^	0.01	0.86	

Note: Distribution of the variable was not normal

* Median (IQR)

^a^ Within-group comparison (Wilcoxon test)

^b^ Between-group comparison (Mann-Whitney u-test)

### Reliability of the appetite questionnaire

To determine the reliability of the questions, 20 patients completed the questionnaire two times with an interval of 14 days. The correlation coefficients of the answers to all the questions were more than 0.7. Analysis of internal reliability also presented satisfactory results (Cronbach’s alpha coefficient = 0.73). The CVR and CVI of all questions were calculated more than 0.8 and the results were satisfactory.

### Side effects

During the two weeks of quadruple therapy concomitant with interventions, 9 patients in the control group and 10 in the *N. Sativa* group reported mild to moderate side effects such as dizziness and headache (4 patients in *N. Sativa* group and 5 patients in placebo group), weakness (4 patients in *N. Sativa* group and 3 patients in placebo group), and bad and bitter taste in their mouth (4 patients in *N. Sativa* group and 4 patients in placebo group) which were gradually resolved. During the next six weeks of the interventions, no adverse effect was observed.

## Discussion

According to our knowledge, this study is the first clinical trial that assesses the effects of consuming 2 gr/day *N. Sativa* powder concomitant with quadruple therapy on appetite and serum ghrelin level in patients with *H. pylori* infection. According to the results of the current clinical trial, consumption of 2 gr/day *N. Sativa* powder for 8 weeks along with quadruple therapy could meaningfully rise the appetite compared to the control group. Serum levels of ghrelin increased in the *N. Sativa* group and decreased in the placebo group, but these changes were not statistically significant. However, it seems that these changes were clinically significant.

It is necessary to mention that in our previous article, we demonstrated that supplementation with 2 gr/day *N. Sativa* powder with quadruple therapy for 8 weeks could significantly rise the eradication of *H. pylori* infection, dietary intake of energy, macronutrients, and most of the micronutrients, body weight, and BMI in the treatment group compared to the placebo [25].

Based on the results of the current study between and within groups of ghrelin levels didn’t change significantly.

The effect of *H. pylori* eradication on ghrelin level is still debated. In some research, eradication of *H. pylori* infection led to an increase or decrease of circulating ghrelin level, while in some other studies, no significant differences in the plasma ghrelin level were observed [26]. For instance, similar to our results, in a clinical trial, the ghrelin concentration in the gastric mucosa (antrum, body, and fundus) and plasma before and after *H. pylori* eradication was evaluated. The findings showed that there were no significant differences between the ghrelin level in the plasma and stomach tissue, before and after *H. pylori* eradication [27]. Also, Isomoto et al. showed that plasma ghrelin concentration, expression of ghrelin immunoreactive cells, and ghrelin mRNA did not change significantly in both eradicated and uneradicated *H. pylori* groups [28]. Equally, in the study of Sook Lee et al., the plasma ghrelin level increased in the treatment group and reduced in the control group after eradication of *H. pylori* infection, but these changes were not statistically significant [29].

In contrast with our results, in the study of Nwokolo et al., the plasma ghrelin concentration increased profoundly in asymptomatic subjects after *H. pylori* eradication [30]. To explain all these discrepancies, it can be stated that although the stomach is the main site of ghrelin synthesis, a significant amount can also be produced in the small intestine and other places in the body [4]. Also, previous studies revealed that the ghrelin levels of *H.pylori*-infected patients were less than non-infected patients. However, the effect of *H. pylori* eradication on the ghrelin concentration is a more complex issue [5, 6], and it seems that some factors such as the grade of gastritis caused by *H. pylori*, duration of the infection, the strain of *H. pylori*, hormonal factors, nutritional changes, etc. may affect the ghrelin level [5, 6, 27].

*H. pylori* secretes some deleterious pathogenic agents into the epithelial cells of the stomach, thereby leading to the malfunctioning of gastric pyloric and oxyntic glands. As a result, the expression of the hormones involved in regulating satiety, hunger, and food consumption such as ghrelin is disrupted [31, 32]. Thus, it can cause loss of appetite and weight in the individuals infected with this infection [32].

Ghrelin is an appetite-stimulating peptide with 28-amino acid primarily produced in the oxyntic gland of the stomach. This neuroendocrine hormone has a well-established part in the regulation of energy homeostasis, fat storage, increasing appetite, food intake [4], and provoking gastric emptying and acid secretion [33]. The effects of ghrelin on stimulating appetite, food consumption, and body weight have been evaluated in several human and animal studies. For instance, in a clinical trial, intravenous ghrelin infusion (5 pmol/kg/in) stimulated the appetite and food intake 30% more than saline infusion [34]. Similarly, ghrelin agonist treatment significantly improves gastric emptying and weight gain in women with anorexia nervosa after four weeks of the intervention [35]. Also, Nakazato et al. in their study indicated that intravenous infusion of anti-ghrelin anti-bodies led to significant weight loss in rats [36].

As we reported in our previous article, 8 weeks’ consumption of 2 gr/day *N. Sativa* powder along with quadruple treatment could significantly rise the *H. pylori* infection eradication in the intervention group compared to the control group [25]. Therefore, the increase of serum ghrelin concentration in the treatment group and the decrease in the control group can be justified by the higher *H. pylori* eradication level in the treatment group in the present study.

In our study, the patient’s appetite improved significantly in the treatment group compared to the placebo after the intervention. Similarly, in the study of Jeong Jang et al. after the eradication of *H.pylori*, the visual analog scales for hunger and prospective food intake were significantly increased [4]. Although the increase in serum ghrelin concentration of the treatment group was not statistically significant in our study, it seems that it was clinically significant and had a positive effect on the patient’s appetite.

On the other hand, there is a strong association between *H. pylori* infection and functional dyspepsia (FD). FD is a common gastrointestinal disorder characterized by early satiation, postprandial fullness, epigastric pain or burning, bloating, nausea, and vomiting with no evidence of organic disease. Although the pathophysiology is not well established, impaired gastric emptying, gastroduodenal motility disorders, visceral hypersensitivity, and psychological abnormality may have a role in the pathogenesis of FD [37]. Since ghrelin affects gastric emptying, secretion, and motility, it may perform a role in the pathophysiology of FD. In other words, the functional disorders in FD may probably disturb the production of ghrelin by the stomach and lead to anorexia and weight loss in some patients [33]. In our previous article, we showed that supplementation with 2 gr/day *N. Sativa* powder along with quadruple treatment led to improve dyspepsia symptoms and increased intakes of energy, macronutrients, and most of the micronutrients. Also, BMI and body weight increased in the *N. Sativa* group in the course of intervention [25]. Thus, the improvement of dyspepsia symptoms may be another good reason for the improvement of appetite.

### Limitations

The small sample size and the short duration of the patient’s follow-up were the limitations of the current study. Measuring more regulatory hormones related to satiety, hunger, and food intake such as leptin, obestatin, and also gastric ghrelin mRNA expression is recommended together with long-term interventions with more sample size in upcoming studies. Moreover, the number of studies that evaluate the patients’ appetite after *H. pylori* eradication is limited and according to our knowledge, the current study is the only clinical trial that evaluated the effect of *N. Sativa* supplementation on appetite in *H. pylori*-infected patients. Therefore, further studies should be considered on this issue.

## Conclusion

This study demonstrated that consumption of 2 gr/day *N. Sativa* powder concurrent with quadruple treatment could improve appetite and elevate the serum ghrelin concentration in *H. pylori-*infected patients. Thus, supplementation with *N. Sativa* powder may be a beneficial adjunctive therapy in these patients.

## Data Availability

The datasets used and analyzed during the current study are available from the corresponding author upon reasonable request.
